# Neurotropic Effects of Cortexin on Models of Mental and Physical Developmental Delay

**DOI:** 10.3390/biomedicines13040860

**Published:** 2025-04-02

**Authors:** Denis V. Kurkin, Dmitry A. Bakulin, Evgeny I. Morkovin, Vladimir I. Petrov, Andrei V. Strygin, Alexey V. Smirnov, Maksim V. Shmidt, Julia V. Gorbunova, Yury A. Kolosov, Olga V. Ivanova, Ivan S. Krysanov, Marina A. Dzhavakhyan, Andrew V. Zaborovsky, Valeria B. Saparova, Igor E. Makarenko, Roman I. Drai, Ilia A. Lugovik, Nikolay A. Verlov, Vladimir S. Burdakov

**Affiliations:** 1 Scientific and Educational Institute of Pharmacy n.a. K.M. Lakin, Russian University of Medicine, Moscow 127473, Russia; strannik986@mail.ru (D.V.K.);; 2 Scientific Center of Innovative Medicines with Pilot Production, Volgograd State Medical University, Volgograd 400131, Russia; 3 Pharm-Holding CJSC, Saint Petersburg 198515, Russia; 4 Petersburg Nuclear Physics Institute Named by B.P. Konstantinov of NRC «Kurchatov Institute», Gatchina 188300, Russia

**Keywords:** cortexin, cerebrolysin, neonatal trauma, rodent model

## Abstract

**Objective:** To evaluate the efficacy of the neurotropic action of cortexin in models of mental and physical developmental delays in rat offspring. **Methods:** The neurotropic properties of bovine brain cortex polypeptides were studied using two models of mental and physical developmental delays in rats: toxic CNS damage (oral administration of ethanol during the last week of pregnancy) and neonatal trauma (ischemia-hypoxia). The drug was administered intramuscularly or rectally as suppositories for 20 days. Treatment efficacy was evaluated using the mNSS scale, open field, rotarod, and adhesive removal tests. A histological examination of the brain was subsequently performed. In a separate series of experiments in mice, the concentration of the test drug cortexin and the reference drug cerebrolysin was determined in blood and brain tissue samples using radioactive iodine (Na125I) labeling of these preparations. **Results:** Modeling developmental delay in rat offspring (due to the toxic effect of ethanol in late pregnancy or neonatal trauma) led to pronounced neurological deficits, manifested by decreased motor activity, and sensorimotor, and coordination disorders. Administration of cortexin in all forms reduced the severity of neurological deficits as measured by mNSS scores, improved motor activity in the Open Field test, enhanced performance in the Adhesive Removal and Rotarod tests, and decreased structural changes in brain tissues. Histological examination revealed reduced neuronal damage in multiple cortical regions, with a significant increase in normal, unchanged neurons compared to placebo groups. Comparison of the blood concentrations of labeled Na125I cortexin depending on the type of administration showed similar distribution profiles in brain tissues, primarily dependent on its blood concentration, which was influenced by the route of administration. **Conclusions:** The results indicate that brain polypeptides (cortexin), administered either intramuscularly or rectally, can reach the systemic circulation and cross the blood-brain barrier, as demonstrated by our distribution studies using radiolabeled preparations. These polypeptides exert comparable neurotropic effects in models of mental and physical developmental delays in offspring caused by neonatal trauma or the toxic effect of ethanol in late pregnancy in rats.

## 1. Introduction

Early brain damage or toxic effects on the central nervous system (CNS) are major etiopathogenetic causes of neurological dysfunction and developmental delays [1]. Specifically, hypoxic-ischemic brain damage during the neonatal period occurs in 8% of births in developed countries and over 25% in developing countries. In many newborns, the consequences of this pathology persist throughout life and are chronic, often irreversible, and difficult to manage with pharmacotherapy. CNS damage due to toxic substances (e.g., alcohol) that may be used knowingly or unknowingly during pregnancy is also a common pathology, presenting equally severe symptoms that significantly impact the individual’s development and quality of life [2]. This highlights the evident need for developing effective pharmacotherapeutic agents to prevent and manage processes underlying CNS damage or developmental defects during fetal or neonatal periods.

In the past few decades, various groups of scientists and pharmaceutical companies have made numerous attempts to develop effective and safe neuroprotective drugs, most of which have not achieved significant results. Notable examples include NXY-059 (a free radical scavenger that failed in the SAINT II trial despite promising preclinical data), tirilazad (a steroid that showed no benefit in stroke patients), selfotel and other NMDA receptor antagonists, and promising anti-inflammatory agents like enlimomab (an anti-ICAM-1 antibody). Nimodipine, a calcium channel blocker initially heralded as a breakthrough for ischemic stroke, failed to demonstrate significant clinical benefit in multiple large-scale trials. Discussions about the feasibility of creating neurotropic agents for treating acute and chronic brain damage continue. However, negative experiences and an increasing body of scientific knowledge about brain organization, physiology, and pathophysiology explain the growing skepticism regarding the possibility of developing such drugs [3].

When considering early-age CNS pathologies, it is crucial to account for the enormous potential of the developing organism, particularly the higher intensity of processes related to neuroplasticity compared to adults. Therefore, the effectiveness of pharmacotherapy for various brain diseases in children may be greater than in adults. In pediatric practice, there are typically specialized preparations with smaller doses, sizes, organoleptic characteristics, and/or dosage forms.

Cortical polypeptides containing neurotrophic factors have been used for several decades to correct acute and/or chronic CNS diseases. Several studies have demonstrated the protective role of such biological preparations in treating brain pathologies caused by acute or chronic cerebral circulation disorders and in preterm infants with a high risk of developmental delay [4,5,6,7].

In this study, we attempted to evaluate the neurotropic effects of cortical polypeptides (cortexin) on rats with experimental models of CNS damage, administered intramuscularly (traditionally) and rectally (more commonly used in pediatrics). According to the literature, based on an analysis of the composition and key physicochemical properties of the cortexin, it has been established that the formulation predominantly consists of 70–95% acidic and neutral polypeptides, with molecular weights ranging from 1000 to 10,000 Da and isoelectric points (pI) of 3.5–9.5 [8,9]. The neurotropic effects of Cortexin may be attributed to the binding of its components to several receptors, including AMPA, kainate, mGluR1, GABAA1, and mGluR5, as demonstrated in our previous in vitro experiment [5].

## 2. Materials and Methods

### 2.1. Ethics Statement

All experiments were conducted in accordance with the legislation of the Russian Federation and the technical standards of the Eurasian Economic Union for Good Laboratory Practice (GOST R 53434-2009, GOST R 51000.4-2011) and Directive 2010/63/EU of the European Parliament and the Council of the European Union. The study protocol was reviewed and approved by the Regional Independent Ethics Committee of the Volgograd Region, registration number: IRB 00,005,839 IORG 0,004,900 (OHRP), protocol No. 024 dated 21 January 2022.

### 2.2. Characteristics of the Test System

The experiment was conducted on the offspring of Wistar rats (*N* = 202) or ICR (CD-1) mice (N = 75), purchased from laboratory animal house Stolbovaya (Moscow oblast, Russian Federation). Upon arrival, the rats were quarantined for 14 days in a separate section of the vivarium. Throughout the experiment, the rats were kept in controlled environmental conditions (20–26 °C and relative humidity 30–70%), with NH3 ≤ 10 mg/m^3^, CO_2_ ≤ 0.15 vol.%, and unlimited access to food and water. The light cycle was 12 h of light and 12 h of darkness. An air exchange regimen was established, ensuring approximately 15 air changes per hour [10]. All painful procedures were performed under general anesthesia using a single intraperitoneal injection of zolazepam 20 mg/kg (Zoletil^®^100, Valdepharm, Val-de-Reuil, France) + xylazine 8 mg/kg (Xyla, Interchemie, Venray, The Netherlands) or inhalation anesthesia. At the end of the experiment, the rats were euthanized in a CO_2_ chamber.

### 2.3. Study Design

**Figure 1:**
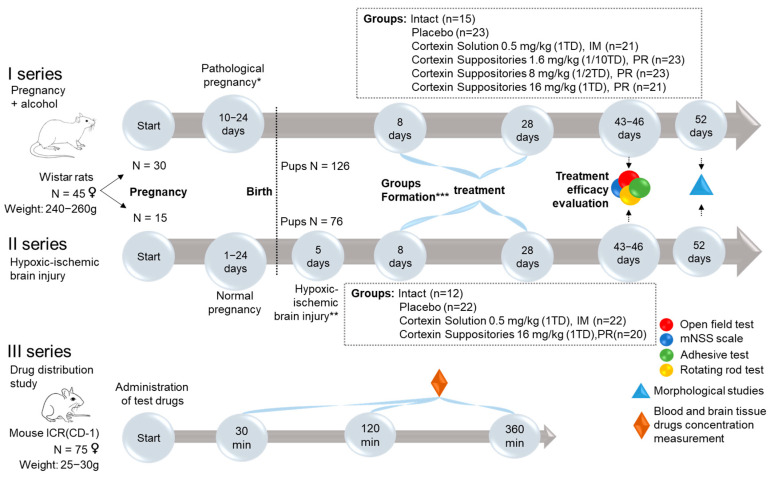
Study design. Note: *—Pathological pregnancy was modeled by ethanol administration during the last week of pregnancy (three administrations of ethanol (2 g/kg) at 48-h intervals); **—Hypoxic-ischemic brain injury was modeled by sequential ischemia (occlusion of the left common carotid artery) and brain hypoxia (exposing the pups for 60 min to a gas mixture of 92% nitrogen and 8% oxygen); ***—The pups were randomly assigned to experimental groups without sex-based division; i.m.—intramuscular administration; PR—per rectum (rectal administration); TD—therapeutic dose.

The overall study design is shown in the figure below (Figure 1).

The first part of the study consisted of two similar series, which included the following stages:

1. Pairing of female rats with males for mating for 1 day (30 females for series 1 and 15 females for series 2).
2. Pregnancy.
3. Modeling of complicated pregnancy (ethanol administration during the last week of pregnancy for series 1, and hypoxic-ischemic brain injury modeling in 5-day-old pups for series 2).
4. Monitoring the rats during the development of the pathology.
5. Formation of groups.
6. Treatment.
7. Evaluation of treatment outcomes.

### 2.4. Modeling Developmental Delay

#### 2.4.1. Modeling Pathological Pregnancy (Administration of Ethanol During the Last Week of Pregnancy)

In series 1, developmental delay in rats was induced by oral administration of a 10% aqueous ethanol solution at a dose of 2 g/kg to females during the last week of pregnancy. Ethanol was administered three times, with a 48-h interval between doses [11,12].

#### 2.4.2. Modeling Hypoxic-Ischemic Brain Injury

In series 2, hypoxic-ischemic brain injury was modeled in 5-day-old rat pups in two stages. First, under inhalation anesthesia, irreversible occlusion of the left common carotid artery was performed. The duration of the surgery did not exceed 60 s. Next, the pups were subjected to hypoxia by placing them for 60 min in an environment containing 8% oxygen and 92% nitrogen, after which they were returned to their home cage [13,14].

### 2.5. Test Objects

The tested drug, cortexin, is a complex of neuropeptides derived from the cerebral cortex of cattle, produced by CJSC “Pharm-Holding” (Saint Petersburg, Russia). In this study, cortexin was tested in two forms: rectal suppositories for children (155 mg) and a lyophilisate for preparing a solution for intramuscular injection (10 mg).

Cortexin in solution form was administered at a therapeutic dose (TD) of 0.5 mg/kg (i.m., hind haunch (gluteus muscle)), and in suppository form at therapeutic doses of 16 mg/kg (1 TD, series 1 and 2), 8 mg/kg (1/2 TD, series 2), or 1.6 mg/kg (1/10 TD, series 2). The suppositories were administered rectally to immature rats in a molten form (t = 37 °C) at appropriate doses based on their body weight, using a dispenser with a plastic probe. The placebo consisted of the melted base substance and was administered in equivalent volumes.

### 2.6. Evaluation of Treatment Efficacy

In both series 1 and 2, the effectiveness of the therapy was determined by evaluating the psychoneurological parameters of the rats (scores on the mNSS scale, motor and exploratory activity in the Open Field test, sensory-motor functions in the Adhesive Removal test, and coordination disorders in the Rotarod test) as well as by analyzing the pathological changes in the brain through histological examination.

#### 2.6.1. mNSS Scale

Neurological deficits in rats were assessed using the mNSS (Modified Neurological Severity Scores) scale. This scale includes tests to identify disorders of motor activity (muscle status and abnormal movements), sensory functions (visual, tactile, and proprioceptive), reflexes, and coordination of movements. Evaluated parameters included: the rat’s motor activity when suspended by the tail; walking patterns on a surface; coordination of movements when walking on a beam; and the strength of reflex responses, including the auricular and corneal reflexes. The maximum score was 14, and the minimum score was 0.

#### 2.6.2. Open Field Test

In this study, we used the “Open Field” apparatus (OpenScience, Moscow, Russia), which is white, circular, and has a floor divided into sectors with holes. During a 3-min test session in the Open Field test, the “number of sectors crossed” was recorded as an indicator of motor activity [15]. Additionally, the “number of holes explored” and counts of “supported rearing” (with wall support) and “unsupported rearing” were documented. These indicators in the Open Field test were interpreted as measures of motor and exploratory activity, characteristic of rodents when exploring a new environment.

#### 2.6.3. Adhesive Removal Test

Fine motor skills and sensitivity of the palmar surface of each forelimb were assessed using the Adhesive Removal test [16]. During the test, square pieces of adhesive tape (5 mm^2^) were placed on the volar surface of the forelimbs. After returning to a cage similar to their home cage, the time taken for the rats to notice the sticker (interpreted as an indicator of sensory function) and the time taken to remove the sticker (interpreted as an indicator of fine motor skills) were recorded over a 3-min period.

#### 2.6.4. Rotarod Test

In the Rotarod test, the latency to fall from a rotating rod (25 rpm) of the apparatus (Neurobotics LLC, Moscow, Russia) over a 3-min period was recorded. This time, compared to the control group without pathology, was interpreted as an indicator of coordination impairment [17].

#### 2.6.5. Morphometric Analysis

The brain was completely extracted from the cranial cavity and sectioned in the frontal plane. The first cut was made at −1.80 mm relative to bregma, the second at −6.80 mm, and the cerebellum along with the brainstem was removed with a third cut. The brain was fixed in 10% neutral buffered formalin and processed according to standard procedures, including dehydration through a graded series of alcohols. For histological preparations, brain fragments were embedded in paraffin using standard methods, and serial frontal sections (4–5 μm thick) were obtained using a rotary microtome; these sections were stained with thionin by the Nissl method.

Brain tissue damage was assessed using standard morphometric methods. The extent of neuronal damage was evaluated by classifying neurons into three groups: normal, unchanged neurons (NN); slightly modified neurons (MN) with preserved nuclei but exhibiting structural or tinctorial changes in cytoplasmic components (e.g., swelling, hyperchromatosis, chromatolysis, or central tinctorial acidophilia); and roughly altered neurons (AN) characterized by significant shrinkage, severe alteration, homogenizing changes, or the presence of shadow cells. The relative numerical density of unchanged neurons and neurons with mild and severe changes was determined. Histological sections were microphotographed using an Olympus digital camera (Olympus Corporation, Tokyo, Japan) coupled with a MICROS microscope (MICROS Austria, St. Veit/Glan, Austria).

Pathological changes were heterogeneous across various brain structures, although the most significant signs of neurodegeneration were observed in the cerebral cortex. To maintain consistency with data obtained from previous experimental studies on the impact of various pathogenic factors on the developing brain, we focused primarily on structural alterations in distinct functional areas of the neocortex (motor, somatosensory, visual, auditory, piriform, and entorhinal cortices). These regions were examined on serial frontal sections at levels ranging from −2.30 mm to −6.30 mm relative to bregma.

### 2.7. Investigation of Drug Distribution

In series 3 (Figure 1), the distribution of cortexin in different forms was assessed in blood and brain tissue after a single administration in ICR (CD-1) mice. Cerebrolysin was used as the reference drug.

The study was conducted on female outbred ICR (CD-1) mice. At the start of the experiment, all mice were clinically healthy and examined by a veterinarian. The mice were housed in groups of 5 per cage with a 12-h light-dark cycle at a temperature of 20–22 °C and were provided with a standard diet. Before the study, the mice were food-restricted. The study was conducted in accordance with the rules for working with laboratory mice and bioethical standards.

Experimental groups were randomly formed: mice receiving cerebrolysin (Cerebrolysin^®^, 2.5 mL/kg, i.m., n = 15); mice receiving cortexin (Cortexin^®^, 16 mg/kg, i.v., n = 15); mice receiving rectal suppositories with the peptide drug in two doses (8 mg/kg and 16 mg/kg, rectally, n = 15). In each group, ICR (CD-1) mice were euthanized at control points 30 min, 2, and 6 h after drug administration. Samples were collected from each mouse—blood and brain. Each sample was weighed, and the accumulated dose was measured. Dose accumulation was measured using a TRI-CARB 5110 TR liquid scintillation alpha-beta radiometer PerkinElmer (PerkinElmer, Waltham, MA, USA) with gamma vials [18].

All tested drugs were iodinated using Na125I according to a standard protocol with chloramine T [19]. To purify the drug from free radioactive markers, gel filtration was performed on a PD MiniTrap G-10 column (Cytiva, Uppsala, Sweden). The radiochemical purity of the prepared drug was controlled using thin-layer chromatography (TLC). Samples of the mixture before addition, after incubation with chloramine T, and after gel filtration were applied to a Silufol TLC plate, using 96% ethanol as the liquid phase. TLC radiography was performed on an Amersham Typhoon 5 laser scanner (Cytiva, Uppsala, Sweden).

### 2.8. Data Analysis

Statistical analysis of the study results was performed using Microsoft Office Excel 2013 (Microsoft, Redmond, DC, USA) and Prism 6 (GraphPad Software Inc., San Diego, CA, USA). In planning our experiments, we aimed to minimize the number of animal groups for ethical considerations. The study involves four factors: the presence of pathology, test drug, administration route, and dose. A complete factorial design would require at least 16 animal groups, which we deemed ethically unacceptable. Therefore, we combined these factors and considered them as a single factor “intervention type.” The Shapiro-Wilk test was used to check for the normality of distribution. Intergroup differences were analyzed using parametric or non-parametric methods depending on the type of distribution: one-way analysis of variance (One-Way ANOVA) or the Kruskal-Wallis rank one-way analysis of variance, followed by post hoc tests (Dunnett’s or Dunn’s test). For normally distributed data, results are described as mean ± standard deviation (mean ± SD). For non-normally distributed data, results are described as medians and interquartile ranges (median [Q1; Q3]). Differences were considered significant at *p* < 0.05.

## 3. Results

### 3.1. Evaluation of Neurological Deficit

#### 3.1.1. Pathological Pregnancy Model (Ethanol Administration During the Last Week of Pregnancy)

**Figure 2:**
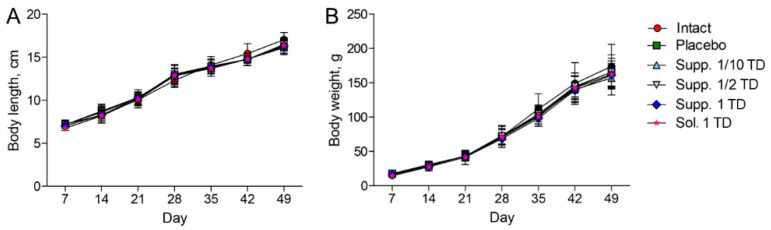
Dynamics of body length (**A**) and body weight (**B**) (pathological pregnancy model). Note: Data shown as the mean ± SD; Sol.—cortexin in solution form; Supp.—cortexin in suppositories form; TD—therapeutic dose.

In experimental series 1, all rats showed consistent increases in body length and weight throughout the experiment; there were no statistically significant differences between the groups (Figure 2A,B).

**Figure 3:**
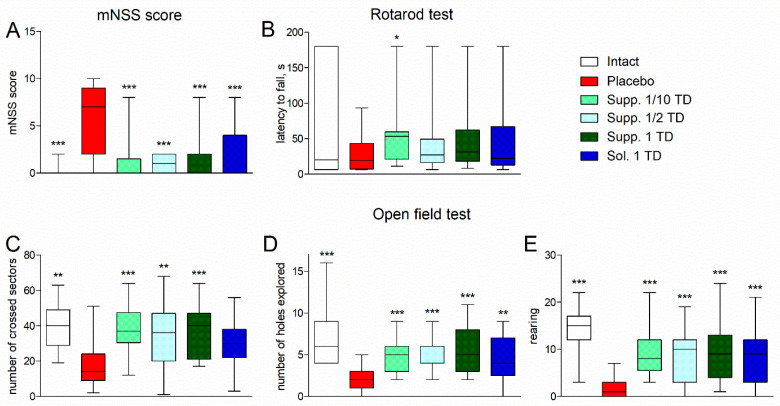
mNSS scores (**A**), latency to fall from a rotating rod (**B**). Scores in the open field test: number of crossed sectors (**C**), number of holes explored (**D**), and rearing (**E**) (pathological pregnancy model). Note: data are shown as median, interquartile range, minimum and maximum values; Sol.—cortexin in solution form; Supp.—cortexin in suppositories form; TD—therapeutic dose; *, **, ***—*p* < 0.05, *p* < 0.01, *p* < 0.001 compared to placebo group (Kruskal-Wallis rank analysis and the Dunn post-hoc test).

Rats in the placebo group exhibited signs of mild or moderate neurological deficits, with a median mNSS score of 7 [2; 9], whereas in the other groups, the median score was 0 points (or 1 point in the group receiving suppositories at a dose of 1/2 of the therapeutic dose) (Figure 3A). Statistically significant differences from the placebo group were observed in both the intact group and the treatment groups (*p* < 0.001). No statistically significant differences were found between the intact group and the treatment groups (*p* > 0.05).

In the Rotarod test, the placebo group showed a tendency towards a reduction in the latency to fall from a rotating rod compared to intact rats (Figure 3B). All treatment groups showed a tendency towards an increase in the latency to fall from a rotating rod. The group receiving cortexin suppositories at a dose of 1/10 of the therapeutic dose showed statistically significant differences (*p* = 0.0177).

The experimental pathology was accompanied by a statistically significant reduction in horizontal locomotor activity in the Open field test: the median number of sectors crossed by rats in the placebo group was 14 [9; 24], while the intact rats had a statistically significantly higher value of 40 [29; 49] (*p* < 0.01) (Figure 3C). In treated rats, this value increased to 30–40. The increase was statistically significant (*p* < 0.001–0.01) in the groups that received cortexin in suppository form.

Exploratory activity (number of holes explored) was also significantly reduced in the placebo group compared to the intact group (2 [1; 3] vs. 6 [4; 9], *p* < 0.001) (Figure 3D). In treated rats, this indicator improved to 4–5, resulting in statistically significant differences from the placebo group (*p* < 0.01–0.001).

Vertical activity (supported and unsupported rearing) in the placebo group was minimal, with a median total of 1 [0; 3] rears compared to 15 [12; 17] in the intact group (Figure 3E). In rats from the other groups, the number of rears was statistically significantly higher than in the placebo group (*p* < 0.001), but lower than in the intact group.

**Figure 4:**
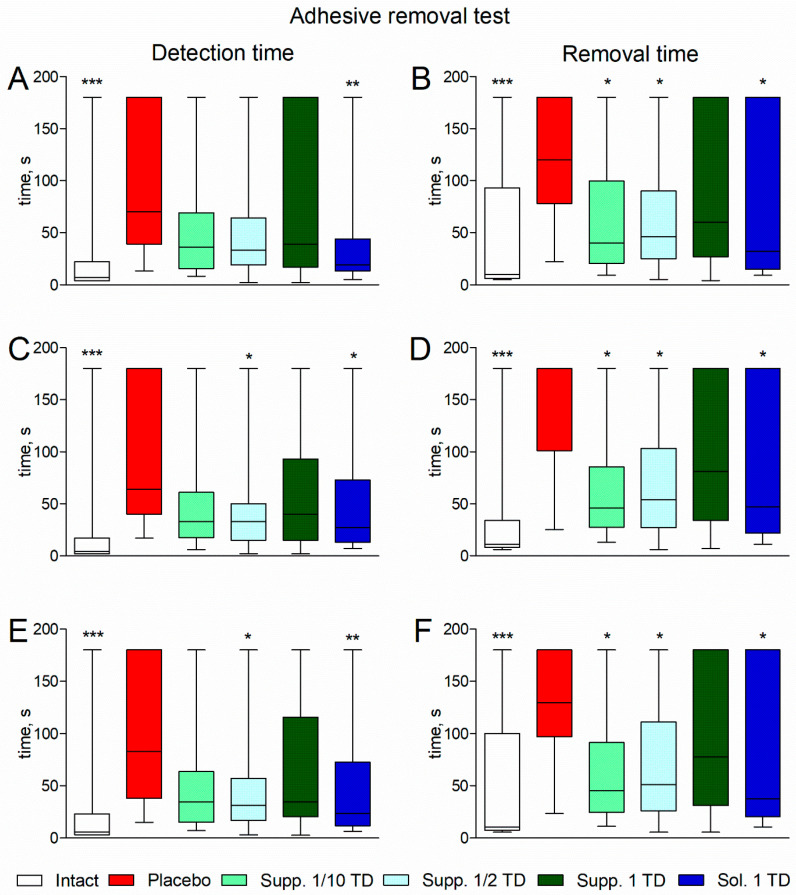
Detection time (**A**) and removal time (**B**) for the sticker from the left paw; detection time (**C**) and removal time (**D**) for the sticker from the right paw; average detection time (**E**) and removal time (**F**) for the sticker from the palmar surface of the forepaws in the adhesive removal test (pathological pregnancy model). Note: data shown as median, interquartile range, minimum and maximum values; Sol.—cortexin in solution form; Supp.—cortexin in suppositories form; TD—therapeutic dose; *, **, ***—*p* < 0.05, *p* < 0.01, *p* < 0.001 compared to placebo group (Kruskal-Wallis rank analysis and the Dunn post-hoc test).

In rats with experimental pathology given a placebo, significant deterioration was observed in the adhesive removal test. The median adhesive tape detection time on the left and right paws was 70 [39; 180] and 64 [40; 180] seconds, respectively (compared to 7 [4; 22] and 4 [2; 17] seconds in the intact group; *p* < 0.001). The median average (between the left and right paws) detection time was 83 [38; 180] seconds (compared to 5.5 [3; 23] seconds in the intact group; *p* < 0.001) (Figure 4, left half of the figure).

The median adhesive tape removal time for the left and right paws was 120 [78; 180] and 180 [101; 180] seconds, respectively (compared to 10 [6; 93] and 11 [8; 34] seconds in the intact group; *p* < 0.001). The median average (between the left and right paws) removal time was 129.5 [97; 180] seconds (compared to 10.5 [7.5; 100] seconds in the intact group; *p* < 0.001) (Figure 4, right half of the figure).

Rats receiving treatment showed improvement in adhesive removal test indicators. Statistically significant differences from the placebo group in detection and removal times were noted in the groups treated with cortexin in solution form and suppositories at 1/10 and 1/2 of the therapeutic dose (*p* < 0.05) (Figure 4).

#### 3.1.2. Hypoxic-Ischemic Brain Injury Model

**Figure 5:**
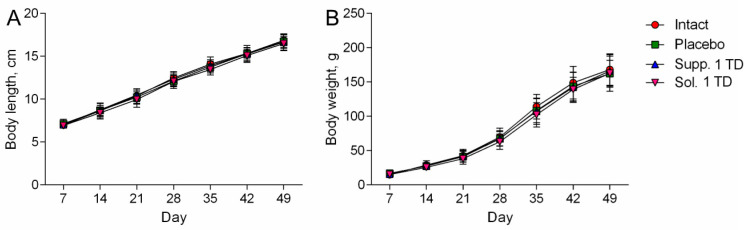
Dynamics of body length (**A**) and body weight (**B**) (hypoxic-ischemic brain injury model). Note: Data shown as the mean ± SD; Sol.—cortexin in solution form; Supp.—cortexin in suppositories form; TD—therapeutic dose.

As in the previous series, all groups of rats showed consistent increases in body length and weight throughout the experiment, with no statistically significant differences between the groups (Figure 5A,B).

**Figure 6:**
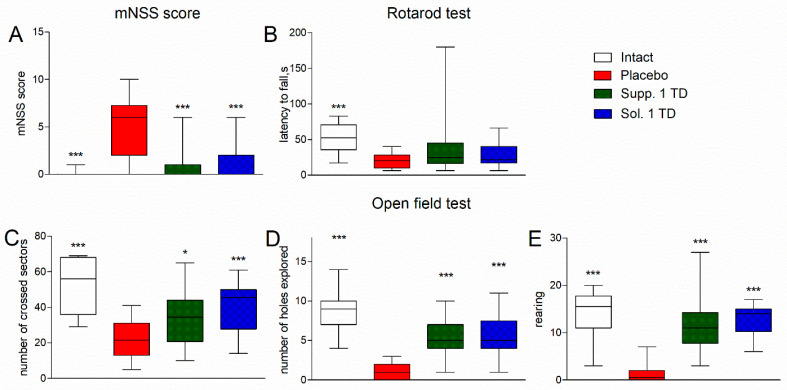
mNSS scores (**A**), latency to fall from a rotating rod (**B**). Scores in the open field test: number of crossed sectors (**C**), number of holes explored (**D**), and rearing (**E**) (hypoxic-ischemic brain injury model). Note: data shown as median, interquartile range, minimum, and maximum values; Sol.—cortexin in solution form; Supp.—cortexin in suppositories form; TD—therapeutic dose; *, ***—*p* < 0.05, *p* < 0.001 compared to placebo group (Kruskal-Wallis rank analysis and the Dunn post-hoc test).

As in the previous series of experiments, rats in the placebo group exhibited signs of moderate neurological deficits, with a median mNSS score of 6 [2; 7.25], whereas in the other groups, the median score was 0 points, which was statistically significantly lower than in the placebo group (*p* < 0.001) (Figure 6A). There were no statistically significant differences between the intact group and the treatment groups (*p* > 0.05).

In this series of experiments, the Rotarod test showed a significant decrease in the latency to fall from a rotating rod in the placebo group compared to intact rats (20 [10; 28.25] vs. 52.5 [35.5; 70.75], *p* < 0.001) (Figure 6B). All treatment groups showed a tendency towards an increase in the latency to fall, but the differences were not statistically significant (*p* > 0.05).

The experimental pathology was accompanied by a significant reduction in horizontal locomotor activity in the Open Field test: the median number of sectors crossed by rats in the placebo group was 21.5 [13; 31], while in the intact rats, the value was statistically significantly (*p* < 0.001) 2.5 times higher, at 56 [36; 68] (Figure 6C). Treated rats showed an increase in this indicator (compared to the placebo group): 34.5 [27.75; 44] (*p* < 0.05) and 45.5 [27.75; 50] (*p* < 0.001) for the test drug in suppository and solution forms, respectively.

Exploratory activity (number of holes explored) was also significantly reduced in the placebo group compared to the intact group (1 [0; 2] vs. 9 [7; 10], *p* < 0.001) (Figure 6D). Treated rats showed a comparable improvement in this indicator (up to 5), leading to statistically significant differences from the placebo group (*p* < 0.001).

Vertical activity (supported and unsupported rearing) in the placebo group was minimal, with a median total of 0.5 [0; 2] rears compared to 15.5 [11; 17.75] in the intact group (Figure 6E). Rats in other (treated) groups had significantly higher numbers of rears, than rats in the placebo group (*p* < 0.001).

**Figure 7:**
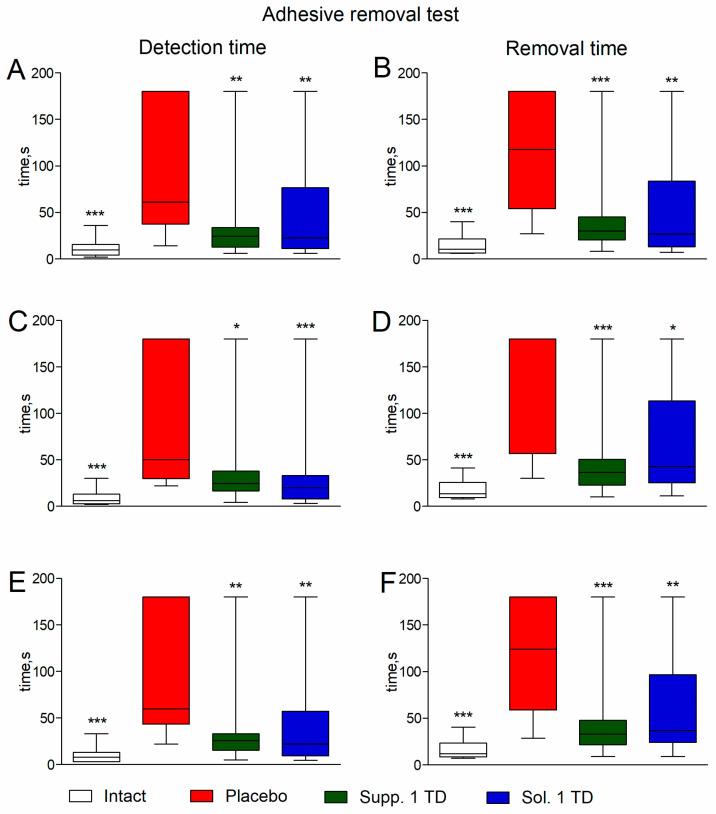
Detection time (**A**) and removal time (**B**) for the sticker from the left paw; detection time (**C**) and removal time (**D**) for the sticker from the right paw; average detection time (**E**) and removal time (**F**) for the sticker from the palmar surface of the forepaws in the adhesive removal test (hypoxic-ischemic brain injury model). Note: data shown as median, interquartile range, minimum, and maximum values; Sol.—cortexin in solution form; Supp.—cortexin in suppositories form; TD—therapeutic dose; *, **, ***—*p* < 0.05, *p* < 0.01, *p* < 0.001 compared to placebo group (Kruskal-Wallis rank analysis and the Dunn post-hoc test).

Rats with experimental pathology given a placebo showed significant deterioration in the adhesive removal test. The median adhesive tape detection time on the left and right paws was 61 [37.5; 180] and 50 [29.5; 180] seconds, respectively (compared to 9.5 [4; 15.5] and 6 [2.5; 13.25] seconds in the intact group; *p* < 0.001). The median average (between the left and right paws) detection time was 59.5 [43.38; 180] seconds (compared to 7.75 [3; 12.88] seconds in the intact group; *p* < 0.001) (Figure 7, left half of the figure).

The median adhesive tape removal time for the left and right paws was 117.5 [54; 180] and 180 [56.75; 180] seconds, respectively (compared to 10.5 [6.25; 21.5] and 13.5 [9.25; 25.75] seconds in the intact group; *p* < 0.001). The median average (between the left and right paws) removal time was 124 [58.75; 180] seconds (compared to 12 [8.5; 23.63] seconds in the intact group; *p* < 0.001) (Figure 7, right half of the figure).

Both treatment groups showed significant improvement in detection and removal times in the adhesive removal test (*p* < 0.001–0.05). In the cortexin suppository and cortexin solution groups, the median average detection time was 25.5 [15.38; 33.13] and 22 [9.375; 57.25] seconds, respectively, and the median average removal time was 33 [21.5; 47.75] and 36.5 [24.13; 96.38] seconds, respectively. These values were significantly lower compared to the placebo group (*p* < 0.001–0.05).

### 3.2. Results of Histologic Examination

#### 3.2.1. Pathological Pregnancy Model (Ethanol Administration During the Last Week of Pregnancy)

**Figure 8:**
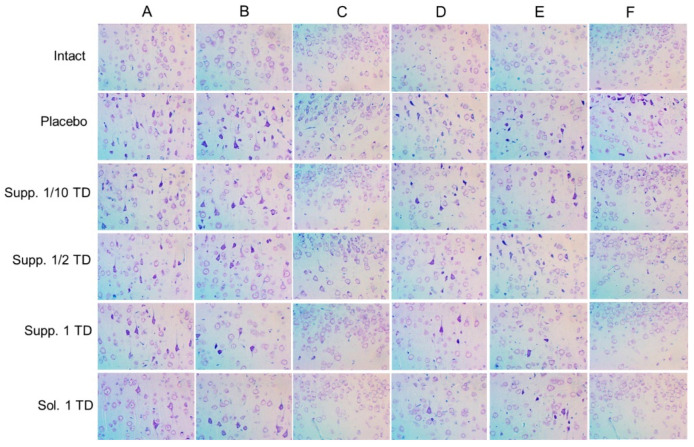
Results of histologic studies of brain samples (pathological pregnancy model). Note: Functional areas of the cerebral cortex: A—motor, B—somatosensory, C—piriform, D—visual, E—auditory, F—entorhinal; Sol.—cortexin in solution form; Supp.—cortexin in suppositories form; TD—therapeutic dose. Microscope magnification: ×400.

The offspring of females exposed to alcohol primarily exhibited mild to moderately pronounced pathomorphological changes in various brain structures. These changes were heterogeneous and most noticeable in different regions of the cerebral cortex, structures of the limbic system, and certain nuclei of the brainstem. Therefore, to ensure continuity with data obtained from other experimental studies on the impact of various pathogenic factors on the developing brain, the main focus was placed on structural changes occurring in various functional areas of the neocortex. Pathological changes in the cerebral cortex were characterized primarily by significant polymorphism. Nonetheless, two main patterns of these changes can be distinguished. The first pattern involved the development of peripheral, central, or segmental chromatolysis of varying degrees, often accompanied by cytoplasmic vacuolization. Typically, multiple small vacuoles were found in the cytoplasm, sometimes merging into larger cavities. The nuclei of such cells were slightly deformed, with increased basophilia of the nucleoplasm. The predominant type of pathomorphologic changes was hyperchromic alterations in neurons and glial cells, characterized by high basophilia and light-optical density of the cytoplasmic and karyoplasmic matrix. These cells were smaller in volume than normal, having elongated rod-like, triangular, or polygonal shapes. In the final stage of this type of damage, it was no longer possible to visualize the pycnotic nucleus against the backdrop of sharply pronounced basophilia of the perikaryon. In the placebo group, the highest number of mildly and moderately altered cells was found in the somatosensory (15.59 ± 2.15%), visual (12.96 ± 1.14%), and auditory (12.97 ± 1.34%) areas of the neocortex. The largest foci of severe hyperchromic changes were noted in the entorhinal cortex, where the number of such cells reached 6.42 ± 4.18% (Figure 8).

In the cortexin solution group (1 TD, 0.5 mg/kg, i.m.), mild pathomorphologic changes predominated in the neocortex structures. Neurons with moderate hyperchromia of the nucleus and cytoplasm and smaller cell bodies were predominantly found in the ganglionic layer. In the entorhinal area of the cortex, most neurons had a structure within the physiological norm, with only a few cells showing mild hyperchromic changes (0.77 ± 0.30%). In the somatosensory cortex, the number of cells with pronounced pycnotic changes was higher than in samples from control rats (placebo). In the motor and entorhinal cortex, the overall numerical density of dystrophically altered neurons was significantly lower, primarily due to the absence of irreversibly damaged cells. In the piriform, visual, and auditory cortex, the number of neurons with moderately pronounced pycnotic changes (0.91 ± 0.26%; 3.25 ± 0.73%; and 6.87 ± 0.78%, respectively) was significantly lower compared to control rats (*p* < 0.05).

In the group that received cortexin in suppository form at 1 TD (16 mg/kg, p.r.), a small number of hyperchromic pyramidal neurons with slightly smaller perikarya, increased cytoplasmic basophilia, and dark pycnotic nuclei were found in various functional areas of the cortex. Single cells with pronounced neurodegenerative changes were detected only in the motor cortex. Morphometric analysis confirmed that rectal administration of cortexin (suppositories) produced a pronounced neurotropic effect. In the somatosensory, piriform, visual, auditory, and entorhinal areas, the number of dystrophically altered neurons was significantly lower compared to the control group (*p* < 0.05). In the motor areas of the neocortex, the pattern of dystrophic changes did not differ significantly from the alcohol fetopathy group without pharmacological correction (placebo).

In the group that received cortexin in suppository form at 1/2 TD (8 mg/kg, p.r.), in all studied functional areas of the cerebral cortex, foci of pycnotic changes in the neurons of the ganglionic layer were found. These cells were characterized by a polygonal or triangular shape and increased cytoplasmic basophilia. In some cases, the cells appeared as intensely stained pycnotic formations with barely visible nuclei. Statistical counting of the proportion of damaged neurons indicated relatively weak neurotropic efficacy of the drug at the specified dose. Essentially, a significant reduction in the number of dystrophically altered neurons was recorded only in the visual areas of the neocortex (7.70 ± 1.58% compared to 12.96 ± 1.14% in the control). It should be emphasized that in other functional areas of the cortex, pathomorphological changes were less pronounced than in the control group rats, but the differences were not statistically significant.

In the brain micropreparations of rats that received cortexin at 1/10 TD (1.6 mg/kg, p.r.), pathomorphological changes were predominantly expressed as the presence of shrunken and hyperchromic cells in the ganglionic layer. Morphometric counting of damaged neurons showed that cortexin in suppository form at a dose of 1/10 exerted moderate neurotropic effects, most noticeably in the piriform cortex. In the motor, somatosensory, and visual cortex, the relative numerical density of dystrophically altered neurons did not differ from the control group (placebo). In the auditory and entorhinal areas, there was a trend (*p* ≥ 0.05) towards lesser severity of neurodegenerative changes.

#### 3.2.2. Hypoxic-Ischemic Brain Injury Model

**Figure 9:**
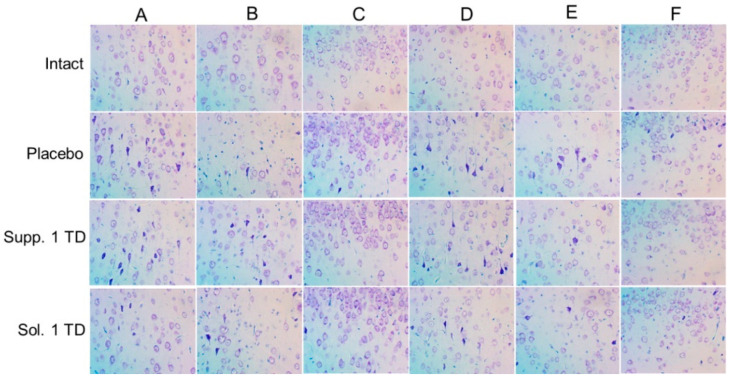
Results of histologic studies of brain samples (hypoxic-ischemic brain injury model). Note: Functional areas of the cerebral cortex: A—motor, B—somatosensory, C—piriform, D—visual, E—auditory, F—entorhinal; Sol.—cortexin in solution form; Supp.—cortexin in suppositories form; TD—therapeutic dose. Microscope magnification: ×400.

In the brain, samples of nearly all rats that had their left carotid artery ligated on the fifth day after birth and were subsequently placed in a low-oxygen environment, foci of cellular depletion were detected. These areas lacked neurons and exhibited focal proliferation of glial cells. This was most frequently observed in the temporal and parietal cortex, with the sizes varying significantly from small fragments of the ganglionic layer to extensive areas affecting all cortical regions. Morphometric analysis showed that various functional areas of the cortex exhibited focal changes with mild and moderate hyperchromic damage to neurons and glial cells. In the placebo group, statistical counting revealed a relatively uniform distribution of such damaged neurons across the examined functional areas: motor—18.18 ± 1.89%; somatosensory—13.29 ± 1.56%; visual—11.40 ± 5.80%; auditory—15.32 ± 2.55%; piriform—9.72 ± 6.11%. Severe hyperchromic changes, associated with pronounced neurodegenerative processes, were observed only in the visual cortex, where the number of such cells reached 7.07 ± 2.54% (Figure 9).

In the somatosensory and auditory cortex of rats that underwent neonatal ischemia-hypoxia and were administered the cortexin in solution form (1 TD, 0.5 mg/kg, i.m.), the number of damaged hyperchromic cells did not significantly differ from the samples in the group without pharmacological correction (11.38 ± 3.20% and 10.18 ± 1.64%, respectively). Mild and moderate pathomorphological changes predominated. The cells had a slightly elongated or polygonal shape, with pycnotic nuclei clearly visualized against the backdrop of weakly basophilic homogeneous cytoplasm. Shrunken, hyperchromic cells were found in the pyramidal cells of the motor and visual areas of the neocortex; however, their relative numerical density was significantly lower compared to the control group samples. In the entorhinal cortex, the number of dystrophically altered cells was lower than in the control (placebo), but the differences were not significant. It should be noted that in rats treated with cortexin, the histological appearance in the piriform areas of the cerebral cortex was similar to that of intact rats.

In the group that received cortexin in suppository form at 1 TD (16 mg/kg, p.r.), from the standpoint of morphological changes in brain structures, the use of cortexin suppositories had a somewhat less pronounced effect compared to the injectable form of the drug. In the motor, somatosensory, and visual areas of the cerebral cortex, mild and moderate pathomorphological changes predominated—smaller neuron and glial cell sizes, hyperchromatosis of the nucleus and perikaryon. The relative numerical density of these moderately altered neurons reached 15.37 ± 5.83% in the motor cortex, 9.50 ± 1.66% in the somatosensory cortex, and 12.69 ± 2.66% in the visual cortex. In these same areas, isolated neurons were observed where the Nissl substance had merged, resulting in intensely stained narrow, angular formations. The highest number of such neurons was found in the visual cortex, 2.06 ± 1.15% (7.07 ± 2.54% in the control; *p* > 0.05). In the auditory cortex, the number of damaged neurons was significantly lower compared to the control group values and amounted to 4.14 ± 1.46%. In the piriform and entorhinal areas of the cortex, the histological appearance of the tissues did not differ from that observed in the intact group.

Thus, in this series of experiments studying the effects of various pathogenic factors on the developing brain, extremely heterogeneous pathomorphological changes affecting various cerebral structures were noted. This heterogeneity can be attributed to the fundamentally different nature of the experimental impacts (cerebral circulation disorder/hypoxia and alcohol embryofetopathy) and, apparently, the varying degrees of adaptive and neuroplastic processes in the growing brains of the rat pups under these experimental conditions. Therefore, to unify and ensure the continuity of the obtained results, we primarily investigated the structural characteristics of different functional areas of the neocortex, where pathomorphological changes were consistently observed under all types of experimental impacts.

The results of the study demonstrate that the most frequent forms of pathomorphological changes in nerve cells were:

1. Chromatolysis of varying degrees is often accompanied by cytoplasmic vacuolization.
2. Hyperchromatosis, in its extreme form, where nerve cells appear as shrunken, dark homogeneous formations with poorly defined nuclei and nucleoli.

Naturally, the effect of pharmacological correction varied significantly depending on the nature of the pathogenic impact and the doses of the drugs used. Nonetheless, it is worth noting that cortexin, administered in various forms (rectal suppositories and lyophilisate for intramuscular injection) and dosages, demonstrated some effectiveness in reducing the severity of neurodegenerative processes.

When analyzing the neurotropic activity of the therapy conducted in cases of alcohol embryofetopathy, we noted approximately equal effectiveness of the therapy with injectable and rectal administration of cortexin at therapeutic doses (0.5 and 16 mg/kg, respectively). Our data indirectly indicate that the neurotropic effect of rectally administered cortexin depends on the dose. The use of suppository forms of cortexin at doses of 1/2 or 1/10 of the therapeutic dose was characterized by a lesser protective effect.

A similar trend was observed in the pharmacocorrection of combined brain damage in rats induced by general hypoxia with ligation of the left carotid artery. Injectable administration of cortexin exerted a pronounced neurotropic effect, which, compared to the placebo group, manifested in a lower number of damaged neurons in most functional regions of the cortex. Rectal administration of cortexin also significantly prevented the development of neurodegenerative changes, especially in the auditory and entorhinal cortex.

**Figure 10:**
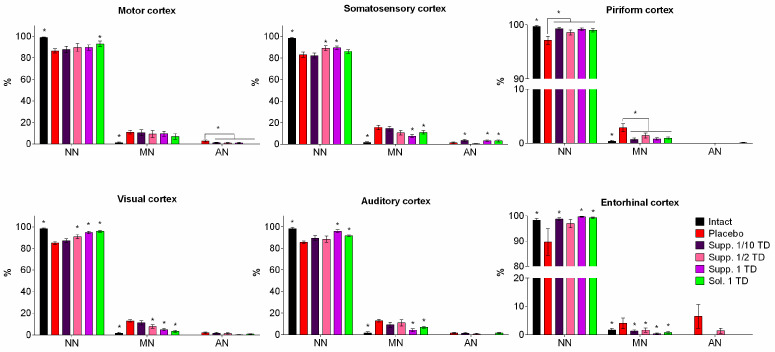
Morphological characteristics of neurodegenerative changes in neurons of the pyramidal layer in various functional areas of the cerebral cortex (pathological pregnancy model). Note: *—*p* < 0.05 compared to placebo group (1-way ANOVA and Dunnett’s post-hoc test). Degree of neuronal damage: normal, unchanged neurons (NN); slightly modified neurons (MN) with preserved nuclei but structural or tinctorial changes in cytoplasmic components (swelling, hyperchromatosis, chromatolysis, central tinctorial acidophilia); roughly altered neurons (AN)—pronounced shriveling, “severe change”, homogenizing change of neurons, shadow cells; data shown as the mean ± SD; Sol.—cortexin in solution form; Supp.—cortexin in suppositories form; TD—therapeutic dose.

**Figure 11:**
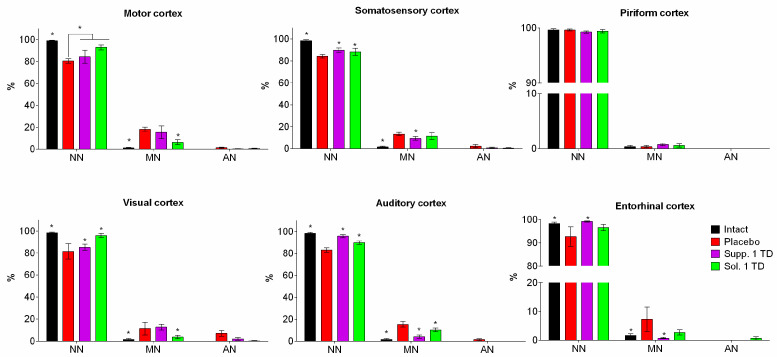
Morphological characteristics of neurodegenerative changes in neurons of the pyramidal layer in various functional areas of the cerebral cortex (hypoxic-ischemic brain injury model). Note: *—*p* < 0.05 compared to placebo group (1-way ANOVA and Dunnett’s post-hoc test). Degree of neuronal damage: normal, unchanged neurons (NN); slightly modified neurons (MN) with preserved nuclei but structural or tinctorial changes in cytoplasmic components (swelling, hyperchromatosis, chromatolysis, central tinctorial acidophilia); roughly altered neurons (AN)—pronounced shriveling, “severe change”, homogenizing change of neurons, shadow cells; data shown as the mean ± SD; Sol.—cortexin in solution form; Supp.—cortexin in suppositories form; TD—therapeutic dose.

Summarizing the obtained morphological data, it can be concluded that under the influence of various negative factors on the developing brain of experimental rats, the use of cortexin, both injectable and rectal, exhibited a pronounced neurotropic effect (Figure 10 and Figure 11).

### 3.3. Determination of the Concentration of the Substances Under Study in Blood and Brain During Intravenous and Rectal Administration

The distribution of cortexin in different forms was assessed in blood and brain tissue after a single administration in ICR (CD-1) mice (Figure 1). Cerebrolysin was used as the reference drug. For cortexin, regardless of the route of administration and dose, the distribution profile of the drug in brain tissues can be considered similar, primarily depending on its concentration in the blood, rather than the route of administration. Additionally, it is notable that the distribution profiles of the peptide drugs cerebrolysin and cortexin (regardless of dose and administration regimen) are similar, with drug concentrations in brain tissues depending on their content in the blood. The maximum concentration of the drugs with intravenous and intramuscular administration was observed at 30 min post-administration, which aligns well with previously obtained data on the distribution of peptide drugs [20]. The concentration of the drug in the blood with rectal administration is characterized by a lower relative decline over time, with the maximum concentration also occurring at 30 min post-administration.

**Figure 12:**
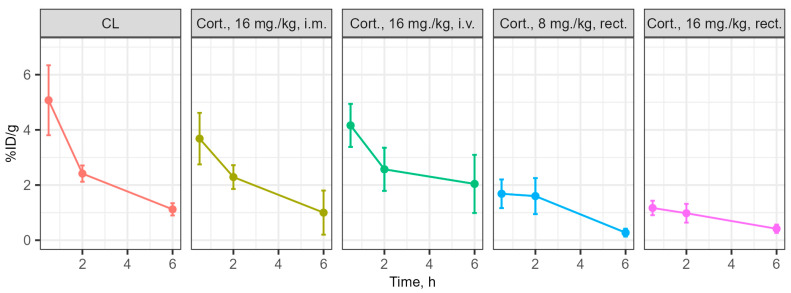
Dynamics of the drug content in the blood at 0.5–2–6 h, percentage of injected dose per gram (%ID/g). Note: CL—cerebrolysin; Cort.—cortexin; i.m.—intramuscular administration; rect.—rectal administration; data shown as the mean ± SD.

**Figure 13:**
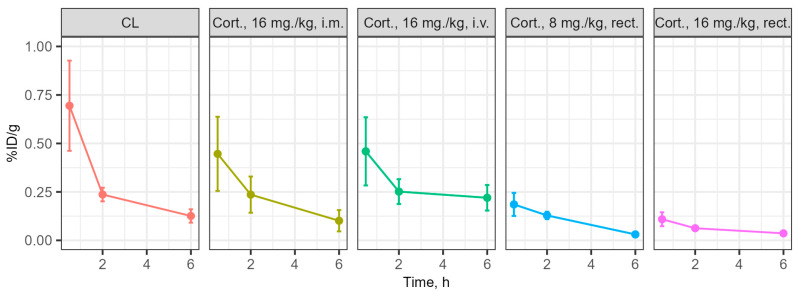
Dynamics of the drug content in the brain at 0.5–2–6 h, percentage of injected dose per gram (%ID/g). Note: CL—cerebrolysin; Cort.—cortexin; i.m.—intramuscular administration; rect.—rectal administration; data shown as the mean ± SD.

Additional analysis showed that the primary route of excretion for the tested drugs is through urine within the first 2 h post-administration. The dynamics of accumulation and the coincidence of the profile of changes in blood concentration of cortexin with rectal administration at doses of 8 mg/kg and 16 mg/kg suggest the presence of a linear dependence of drug absorption in the rectum on the concentration/amount of the administered suppository form of the drug. Thus, by varying the administered dose of the drug during rectal administration in suppository form, it is possible to achieve the required concentration of the drug in the blood and, consequently, in the brain (Figure 12 and Figure 13).

## 4. Discussion

In this study, we investigated the neurotropic effects of cortexin, a polypeptide derived from the cerebral cortex of cattle (bulls), on rat offspring with experimentally induced mental and physical developmental delays. We used two widely used models: (1) pathological pregnancy (ethanol administration in late pregnancy; series 1) [11,12]; and (2) neonatal hypoxic-ischemic brain injury (series 2) [13,14]. Cortexin was administered intramuscularly (i.m.) as a lyophilisate or rectally as suppositories (in the form of melted suppository mass). The results of treatment were evaluated using behavioral tests and histological analysis of brain structures. In a separate series of experiments on mice, we determined the concentration (drug distribution study) of the test drug cortexin or the reference drug cerebrolysin labeled with radioactive iodine (using Na125I [19]) in blood and brain samples.

This study demonstrated that cortexin, in the form of a solution for intramuscular injection or suppositories for rectal administration, exerts a pronounced neurotropic effect in rodent models of developmental delay. The results confirm the potential use of rectal administration as an alternative to parenteral routes, especially in pediatric practice, where minimizing invasive procedures is important [21,22].

Our experimental models induced developmental delays through two different etiological factors—toxic (prenatal alcohol exposure) and hypoxic-ischemic (mimicking neonatal trauma). In both models, symptoms of significant neurological deficit were observed—motor and/or coordination disorders, and sensorimotor dysfunction—corresponding to clinical manifestations observed in humans with developmental disorders caused by similar factors [12,14].

Administration of cortexin in any dosage form significantly improved the condition of the animals. Scores on the mNSS scale and results in the Open Field, Rotarod, and Adhesive Removal tests, which reflect the restoration of motor activity, coordination, and sensorimotor function, were higher in animals that received cortexin. Using this complex of methods for assessing neurological deficit, we demonstrated that cortexin administration significantly reduced indicators of neurological impairment in both experimental series.

The reduction in psychoneurological deficit during treatment is consistent with the results of histological examination. This suggests that the neurotropic effects of cortexin and its impact on CNS functions may be mediated by its ability to affect neurons forming morphological structures. The reduction of hyperchromia and chromatolysis of neurons in cortical regions, especially in the somatosensory and entorhinal areas, indicates that cortexin promotes cell survival.

Our results are consistent with studies demonstrating the neuroprotective potential of cortical polypeptides in developing and acquired brain injuries. For example, cerebrolysin (the reference drug in this case) effectively improved cognitive outcomes in premature infants [7] and stroke patients [4]. However, unlike cerebrolysin, which is administered by injection, our work emphasizes the possibility of rectal administration of cortical polypeptides (using cortexin as an example), expanding the possibilities for their use, for instance in pediatrics.

Our distribution studies using radioactively labeled drug forms provide critical insight into their ability to reach target tissues. The data confirm that polypeptides in cortexin cross the blood-brain barrier following both intramuscular and rectal administration, with brain concentration primarily dependent on blood concentration. This finding is very important as it confirms the biological plausibility of cortexin’s effects on brain tissue regardless of the route of administration.

Additionally, we observed similar distribution profiles for cortexin and cerebrolysin. The linear relationship between rectal dose and blood concentration indicates the possibility of achieving predictable systemic effects with this form of drug administration. Such dose-proportional absorption is advantageous for adapting therapy to pediatric patients, where accurate dosing is of paramount importance.

The demonstrated efficacy of rectally administered cortexin has important implications, as it may serve as a basis for further development and research of this drug or related agents in optimizing administration routes and creating new dosage forms, including for pediatrics. Rectal administration has several advantages over intramuscular injection, including less pain and discomfort, reduced frequency of phobias about visiting medical facilities, lower risk of infection, and the possibility of home administration by caregivers [23]. These benefits are particularly important for children, including those with developmental delays, who already undergo numerous medical procedures and may have increased sensitivity to pain or developed a fear of medical institutions and/or personnel [21,22].

Our results suggest that rectal suppositories could provide a practical alternative to traditional injectable forms of cortexin, potentially improving treatment adherence and patient comfort without compromising therapeutic efficacy. This could expand access to therapy with such drugs, especially in outpatient settings where parenteral administration may be less feasible. Moreover, the dose-dependent effect observed with rectal administration provides a rationale for implementing individualized dosing strategies in clinical practice. Starting with lower doses and titrating based on effect could minimize potential adverse effects, thus optimizing pharmacotherapy for CNS diseases or pathogenic factors leading to developmental delay.

Future research should focus on several areas: (1) elucidating the specific molecular mechanisms underlying the neurotropic effects of cortexin (whether through effects on neurotrophic factors, anti-inflammatory actions, or other pathways); (2) optimizing dosing regimens for rectal administration and developing sustained-release preparations; (3) evaluating long-term outcomes after cortexin treatment, particularly cognitive and behavioral development; (4) identifying the key target and key peptide determining the protective effect, followed by its study and creation of a mono-targeted drug.

To expand the possibilities for using the drug in pediatric clinical practice, careful pharmacokinetic studies in children will be required to establish dosage equivalence between rectal and parenteral preparations. Additionally, patient-centered outcomes should be included in clinical evaluations, including the acceptability of rectal administration and its impact on quality of life.

## 5. Conclusions

Our findings indicate that cortexin exerts comparable neurotropic effects in rodent models of developmental delay when administered either intramuscularly or rectally. Both the suppository and injectable forms significantly reduced neurological deficits, improved performance in the Open Field test, and ameliorated fine motor skill impairments in models induced by toxic damage during late pregnancy and neonatal ischemia-hypoxia. Histological analysis further confirmed that cortexin treatment, irrespective of the administration route, markedly mitigated neurodegenerative changes compared to placebo. Moreover, the pharmacokinetic evaluation revealed a linear relationship between the dose of the rectal suppository and the resulting concentrations in blood and brain, suggesting that the rectal route can reliably achieve therapeutically effective levels. Overall, these results support the potential of cortexin suppositories as a promising alternative to parenteral administration, particularly offering significant advantages for pediatric applications, and warrant further clinical evaluation for the treatment of developmental delays.

### Study Limitations

The results of this preclinical study should be interpreted with caution in clinical settings. The applied generally recognized models of hypoxia were reproduced after birth, whereas in humans hypoxia occurs before or during birth. Systemic exposure of the active ingredient was not determined during the study due to lack of availability. In the study, the dosage form was dosed by body weight, which is not possible in a clinical setting.

Another limitation of the study is the use of immature rats of both sexes, i.e., without separation by sex. The breeding of adult rats was performed as part of the experiment, and excluding offspring by sex would have significantly increased the number of rats required and would have led to the euthanasia of excluded pups. This would be contrary to ethical principles for the treatment of vertebrate rats in scientific experiments. Therefore, the results were analyzed without separating immature rats by sex, based on the assumption that there were no significant differences related to the effects of sex hormones in immature rats during the first 4 weeks of postnatal development. Meanwhile, there were no statistically significant differences in the indicators determined in the experiments within the groups when they were divided by sex.

## Data Availability

Data is available in a public repository: https://doi.org/10.6084/m9.figshare.25909201 (accessed on 9 February 2025).

## Abbreviations

The following abbreviations are used in this manuscript:

AN	Roughly altered neurons
CNS	Central nervous system
MN	Slightly modified neurons
mNSS	Modified Neurological Severity Scores
NN	Unchanged (normal) neurons
PR	Per rectum (rectal administration)
SD	Standard deviation
TD	Therapeutic dose
TLC	Thin-layer chromatography
